# Identification of Novel Drug Candidate for Epithelial Ovarian Cancer *via In Silico* Investigation and *In Vitro* Validation

**DOI:** 10.3389/fonc.2021.745590

**Published:** 2021-10-21

**Authors:** Dan Zou, Jin Bai, Enting Lu, Chunjiao Yang, Jiaqing Liu, Zhenpeng Wen, Xuqin Liu, Zi Jin, Mengdan Xu, Lei Jiang, Ye Zhang, Yi Zhang

**Affiliations:** ^1^ The First Laboratory of Cancer Institute, The First Hospital of China Medical University, Shenyang, China; ^2^ Department of Medical Oncology, Cancer Hospital of China Medical University, Liaoning Cancer Hospital & Institute, Shenyang, China; ^3^ Department of Oncology, Shanghai East Hospital, Tongji University School of Medicine, Shanghai, China; ^4^ Department of Gynecology, First Hospital of China Medical University, Shenyang, China; ^5^ The First Department of Oncology, Shenyang Fifth People’s Hospital, Shenyang, China

**Keywords:** epithelial ovarian cancer, CDK1, piperlongumine, molecular docking, cell cycle

## Abstract

Epithelial ovarian cancer (EOC) has a poor prognosis and high mortality rate; patients are easy to relapse with standard therapies. So, there is an urgent need to develop novel drugs. In this study, differentially expressed genes (DEGs) of EOC were identified in The Cancer Genome Atlas (TCGA) and Gene Expression Omnibus (GEO) databases. Enrichment and protein–protein interaction (PPI) analyses were performed. The drug candidate which has the possibility to treat EOC was predicted by Connectivity Map (CMAP) databases. Moreover, molecular docking was selected to calculate the binding affinity between drug candidate and hub genes. The cytotoxicity of drug candidates was assessed by MTT and colony formation analysis, the proteins coded by hub genes were detected by Western blots, and apoptosis analysis was evaluated by flow cytometry. Finally, 296 overlapping DEGs (|log 2 fold change|>1; q-value <0.05), which were principally involved in the cell cycle (p < 0.05), and cyclin-dependent kinase 1 (CDK1) were screened as the significant hub gene from the PPI network. Furthermore, the 21 drugs were extracted from CMAPs; among them, piperlongumine (PL) showed a lower CMAP score (-0.80, -62.92) and was regarded as the drug candidate. Furthermore, molecular docking results between PL and CDK1 with a docking score of –8.121 kcal/mol were close to the known CDK1 inhibitor (–8.24 kcal/mol). Additionally, *in vitro* experiments showed that PL inhibited proliferation and induced apoptosis *via* targeting CDK1 in EOC SKOV3 cells. Our results reveal that PL may be a novel drug candidate for EOC by inhibiting cell cycle.

## Introduction

Epithelial ovarian cancer (EOC) is an aggressive malignancy and is most frequently diagnosed at an advanced disease stage (1). Currently, the most common treatment is surgery combined with platinum-based combination chemotherapy (2). However, 60% of patients relapsed after first-line therapy (3), and 50% showed resistance to chemotherapy. It is generally believed that EOC chemotherapy resistance is involved in the DNA damage response (DDR) process of the cell cycle (4, 5), in particular single-strand DNA break repair by poly ADP-ribose polymerase (PARP) and double-stranded repair through homologous recombination repair (HRR) of the BRCA1/2 genes. Therefore, it is the key to discovering drugs that affect the mechanism of drug resistance.

Currently, for the treatment of ovarian cancer, agents that target certain stages of the cell cycle, such as cyclin-dependent kinase inhibitors (CDKIs), have shown good efficacy in clinical trials. For example, ribociclib (6), a CDK4/6 inhibitor, which acts on the G1 phase of the cell cycle has been approved by the US Food and Drug Administration (FDA) for the treatment of breast cancer; it has also been used to treat ovarian cancer in phase II clinical trials (NCT02657928). Furthermore, AZD5438 (7, 8), a CDK1/2 inhibitor, enhances radiosensitivity of non-small cell lung cancer by impairing HRR of double-stranded breaks (DSBs) and has already been tested in the preclinical stage. Thus, it has been suggested that targeting the cell cycle is a novel and effective method to treat tumors. However, only a few CDKIs have so far been developed to treat ovarian cancer; thus, more chemotherapeutic agents, which are more efficacious and safe, need to be developed for treating ovarian cancer.

High costs necessitating strong financial support, long timelines, and requirement of substantial resources make development of a novel drug a difficult venture. Drug repurposing is an unconventional approach to identify novel indications of an approved or experimental drug (9). There are several successful examples. For instance, thalidomide was originally developed as an antiemetic in pregnancy but has currently garnered a huge market for the management of multiple myelomas (10). Metformin, widely used for first-line therapy of type 2 diabetes, has been found to possess an additional anticancer property (11). Therefore, repurposing of known drugs is a feasible drug development strategy.

In the present study, by focusing directly on EOC datasets, we aimed to develop a new drug to treat EOC by integrated bioinformatics and *in vitro* experiments. It was principally based on the Connectivity Map (CMAP) databases, which connect genes, drugs, and diseases by numerous cell line experiments. Then, molecular docking was used to matching potential drugs and screened proteins. Furthermore, *in vitro* experiments were used to validate our prediction. To a certain extent, our study may provide a basis for the treatment of EOC.

## Materials and Methods

### Data Source and DEG Acquisition

Epithelial ovarian cancer-related mRNA data of cancer tissues and normal tissues were integrated from RNA-seq data and microarray expression datasets. First, the RNA-seq data were separately collected from The Cancer Genome Atlas (TCGA) database (http://cancergenome.nih.gov/) and the Genotype-Tissue Expression (GTEx) project (https://gtexportal.org/home/); the edition of the TCGA dataset on EOC was updated on July 20, 2019. The microarray expression datasets were obtained from the GEO database (https://www.ncbi.nlm.nih.gov/geo/), and the two gene expression profiles (GSE14407 and GSE54388) were both selected with the GPL590 platform. GSE14407 and GSE54388 were updated on March 25, 2019. Then, differential analyses of the two ways datasets were used by the R package Limma (https://bioconductor.org/packages/release/bioc/html/limma.html) (12) to determine differentially expressed genes (DEGs) with the criteria of |log2(FC)| > 1 and adjusted p-value < 0.05. Furthermore, the differential analysis of RNA-seq data were log2(TPM+1) transformed and analyzed by the Gene Expression Profiling Interactive Analysis (GEPIA) (http://gepia.cancer-pku.cn/) (13).

### Functional Enrichment Analyses

The potential mechanisms of the genes selected were studied, which were imported from the online bioinformatics database Metascape (http://metascape.org/) (14), including gene ontology (GO) Biological Processes and Kyoto Encyclopedia of Genes and Genomes (KEGG) pathway enrichment analyses as well as Protein Protein Interaction (PPI) establishment. In this study, significant terms met the criteria of a p value < 0.05 and the number of enriched genes ≥ 3.

### PPI Network Module Analyses and Identification of Hub Genes

To visualize and analyze the PPI network, we used Cytoscape (version 3.7.0) software (http://www.cytoscape.org/) (15). First, molecular modules were analyzed by Molecular Complex Detection (MCODE) (16) plugin of Cytoscape. The parameter settings were set to default. The criteria were set as follows: MCODE scores > 3 and number of nodes > 4. Next, hub genes were screened from the PPI network using CytoHubba (17) plugin of Cytoscape with the recommended maximal clique centrality (MCC) ranking methods. The top 10 genes were noted as hub genes.

### EOC-Associated Drug Prediction and Gene Set Enrichment Analysis (GSEA)

The connectivity map, which aims to connect the genes, drugs, and disease states by querying the gene lists of upregulated and downregulated genes, was employed. A so-called connectivity score was estimated to assess the priority of the prediction; a positive score denotes a stimulant effect of a drug on the query signatures, whereas a negative score implicates a repressed effect of a drug on the query signatures. This was based on different data and algorithms. CMAP (18), the first-generation connectivity map platform, using a microarray platform (Affymatrix HT_HG_U133A with 22 283 probe sets), screened 1,309 FDA drugs treated in five cell lines, and the connectivity score was from –1 through 1. Then, LINCS (19), the next-generation connectivity map, including 476,251 genome-wide expression signature expression profiles gathered 27,927 perturbagens stimulated by 72 cell lines from 1.3M L1000 profiles. The connectivity score was from –100 through 100. In addition, to investigate the pathways affected by small molecule drugs, the raw data were selected from the CMAP database and analyzed using the function of GSEA from clusterProfiler package (20, 21) and the criteria of a p-value < 0.05, FDR <0.25.

### Molecular Docking Between Drug Candidate and Hub Gene of EOC

The crystal structures of proteins coded by the hub gene were retrieved from the RCSB Protein Data Bank (PDB) (www.rcsb.org/pdb/home/home.do). Moreover, the three-dimensional structure of drugs was searched from PubChem (https://www.ncbi.nlm.nih.gov/pccompound). The molecular docking process involved preparing the proteins and ligands, setting up a grid, and docking the compounds; these were accomplished using the Schrodinger Glide docking protocol (Schrödinger, LLC, NY, USA) (22). The best pose was picked out by the docking score and the rationality of molecular conformation.

### *In Vitro* Cell Lines and Chemicals

Human ovarian cancer cell lines SK-OV-3, CA-OV-3, and HO-8910 were obtained from the Cell Bank of Type Culture Collection of the Chinese Academy (Shanghai, China). SK-OV-3 was cultured in McCoy’s 5A Complete Medium (Thermo Fisher, Belgium). CA-OV-3 was cultured in DMEM medium, and HO-8910 was cultured in RPMI-1640 medium. All the cell lines were cultured in medium supplemented with 10% fetal bovine serum (Greiner Bio-One, Belgium) and antibiotics (penicillin/streptomycin, 100 U/ml, Beyotime, Beijing, China) at 37°C in 5% CO_2_.

PL was purchased from NeOnc Technologies, Inc. (Los Angeles, CA, USA) and diluted with DMSO to make stock solutions of 10 mM. In all cases of cell treatment, the final DMSO concentration in the culture medium never exceeded 0.5%. Stock solutions of all drugs were stored at −20°C.

### Cell Viability Assay

The EOC cell lines were plated to 5 × 103 cells/well in 96-well plates for 24 h, then treated with the indicated concentrations of PL. Next, 50 μl of the MTT reagent (5 mg/ml) was added for 3 h, and then 150 μl of DMSO was admixed to dissolve the formazan crystals. Absorbance was measured at 570 nm using a spectrophotometer (Bio-Rad, Temse, Belgium). Cell viability was determined by dividing the absorbance values of treated cells with that of untreated cells.

### Colony Formation Assay

Depending on the cell line, 200 cells were implanted in each well of a six-well plate and exposed to the indicated concentrations of PL for 24 h. Following this, drugs were withdrawn and cells were grown in normal culture for 14 days. Next, the cells were fixed with acetic acid-methanol (1:4) and stained with diluted crystal violet (1:30). Colonies that consisted of more than 50 cells were counted and calculated. The colony formation efficiency was calculated with the following formula: Survival Fraction = Colonies/Cell numbers × 100%. Three independent experiments were carried out.

### Detection of Apoptotic Cells

Apoptosis was evaluated by using Annexin V-FITC Apoptosis Detection Kit (BD Biosciences Pharmingen, San Diego, CA, USA) according to the manufacturer’s instructions. EOC cell lines (2.5–4.5 × 10^5^ cells/well) were seeded in a six-well plate and grown to 70% confluence. After being treated with various concentrations of PL for 24 h and 48 h, the cells were trypsinized, collected, and washed twice with phosphate-buffered saline (PBS) and stained with FITC-Annexin V and propidium iodide (PI) for 15 min in the dark. The stained cell populations were determined using a FACSCalibur flow cytometer (Becton Dickinson, Bedford, MA, USA), and the data were analyzed using FlowJo Software 7.6 (TreeStar, Inc., San Car-los, CA, USA). Three independent experiments were carried out.

### Western Blots

Cells were extracted, and protein was quantified as described previously (23). The cells were washed twice with PBS, lysed in lysis buffer (1% Triton X-100, 50 mM Tris–HCl pH 7.4, 150 mM NaCl, 10 mM EDTA, 100 mM NaF, 1 mM Na_3_VO_4_, 1 mM PMSF, 2 µg/ml aprotinin), and quantified using a BCA protein quantification kit (cat. no. ab102536; Abcam). The cell lysates were separated by 8% or 15% SDS-PAGE, and the samples were transferred onto a nitrocellulose membrane (Immobilon-P, Millipore; Merck KGaA). After blocking with 5% evaporated skimmed milk in Tris-buffered saline Tween-20 (TBST) buffer (10 mM Tris–HCl pH 7.4, 150 mM NaCl, 0.1% Tween-20) at room temperature for 1 h, primary antibodies were probed and incubated overnight at 4°C. Following three washes with TBST buffer, the membrane was incubated with secondary goat anti-rabbit and goat anti-mouse antibodies for 30 min at room temperature. Finally, the protein bands were detected with enhanced chemiluminescence reagent (SuperSignal™ Western Pico Chemiluminescent Substrate; Pierce; Thermo Fisher Scientific, Inc.) and scanned using the Electrophoresis Gel Imaging Analysis System (DNR Bio Imaging Systems, Neve Yamin, Israel).

### Statistical Analysis

Statistical significance was evaluated with data from at least three independent experiments. GraphPad Prism 7.00 (GraphPad Software, San Diego, CA, USA) was used for data analysis. Statistical analysis was carried out using the Student’s t-test for two groups, as well as one-way ANOVA for more than two groups. Data were presented as the mean ± SD. For all statistical tests, significance was established at p < 0.05. The number of asterisks in the figures indicates the level of statistical significance: *p < 0.05, **p < 0.01, ***p < 0.001, ****p < 0.0001.

## Results

### Identification of Overlapping DEGs for EOC

**Figure 1:**
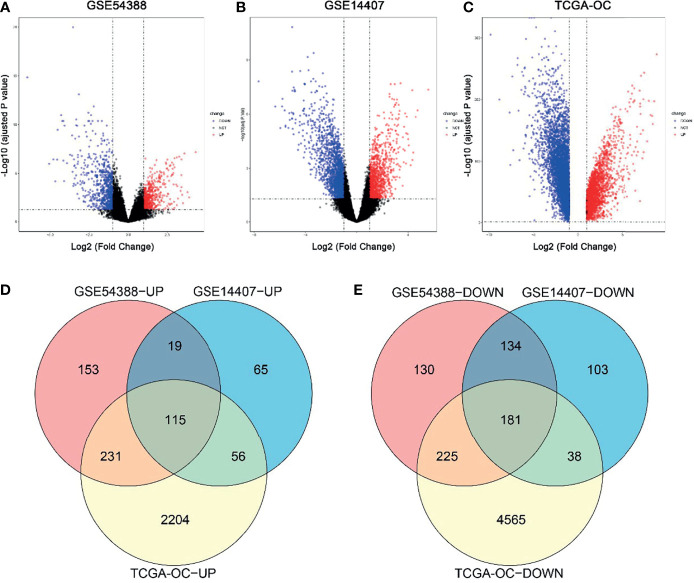
Identification of common differentially expressed genes (DEGs) in three independent datasets. **(A–C)** Volcano plot for the DEGs in GSE54388 **(A)**, GSE14407 **(B)**, and TCGA-OC **(C)** datasets when comparing epithelial ovarian cancer (EOC) to normal ovarian subjects. The x-axis represents log2 (fold change), and y-axis represents significant difference expressed as −log10 (adjusted p-value). DEGs were determined using the limma package. The gene with the adjusted p-value < 0.05,log2FC| ≥ 2 was considered significant. **(D, E)** A total of 115 common upregulated genes **(D)** and 181 common downregulated genes **(E)** were shared between these three independent datasets.

In this study, DEGs and their significant biological characteristics were identified based on various GEO mRNA microarray datasets (GSE14407 and GSE54388) and the TCGA mRNA-seq dataset by integrated bioinformatics analysis of EOC (**Figure 1**). There were a total of 560 samples, including 454 EOC tissues and 106 normal tissues (GSE54388: 16T/6N; GSE14407: 12T/12N; RNA-seq: 426T(TCGA)/88GTEx, respectively). After gene expression assays and data processing and normalizing, we screened DEGs among each mRNA data set using the Limma with the criteria of |log2(FC)| > 1 and an adjusted p-value < 0.05. Overall, a total of 1,188 DEGs were screened from the GSE54388 data set, including 518 upregulated and 670 downregulated genes (**Figure 1A**). There were 711 DEGs, including 255 upregulated and 456 downregulated genes in GSE14407 (**Figure 1B**). Additionally, 7,615 DEGs were selected from the TCGA data set, including 2,606 upregulated and 5,009 downregulated genes (**Figure 1C**). To confirm the reliability of DEGs in EOC, we obtained overlapping DEGs of the three datasets, including 115 common upregulated genes and 181 common downregulated genes (**Figures 1D, E** and **Supplementary Table 1**).

### Functional Enrichment Analyses

**Figure 2:**
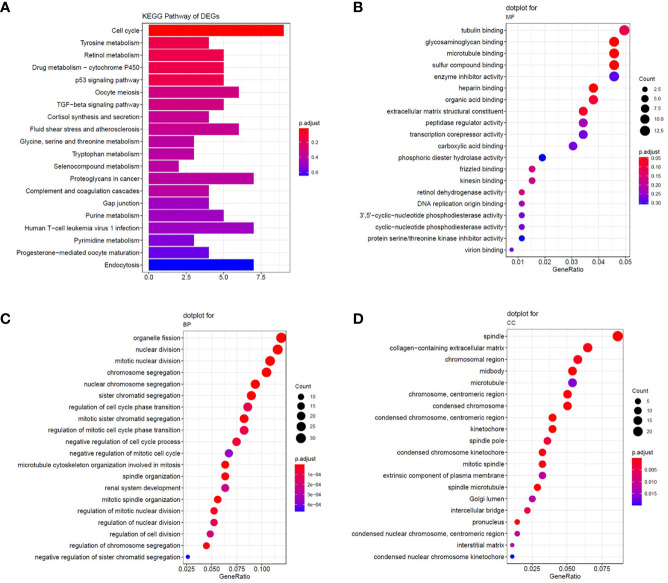
The enrichment analysis of common DEGs. **(A)** Significantly enriched Kyoto Encyclopedia of Genes and Genomes (KEGG) pathways. **(B–D)** The results of GO enrichment for DEGs. Enrichment analysis of the common DEGs was assessed by the Metascape database separately. P-value < 0.05 was considered statistically significant.

We selected overlapping DEGs to investigate the enrichment of EOC in GO and KEGG pathways in Metascape. First, the KEGG pathways for DEGs were mainly cell cycle, oocyte meiosis, and p53 signaling pathway, which were found to be related to the development of multiple tumors and were involved in EOC tumorigenesis and pathogenesis (**Figure 2A**). For GO_BP enrichment analysis, they were enriched in cell cycle and apoptosis, such as cell division, mitotic nuclear division, and mitotic sister chromatid segregation (**Figure 2B**). For GO_MF analysis, they were enriched in DNA replication origine binding and microtubule binding (**Figure 2C**). For GO_CC analysis, they were enriched in spindle, chromosomal region, microtubule, and so on. These results indicated that DEGs might be related to the cell proliferation process (**Figure 2D**). In addition, the KEGG pathways of downregulated DEGs were enriched in tyrosine metabolism, drug metabolism-cytochrome P450, and retinol metabolism (**Figure 2A**).

### PPI Network Module Analyses and Identification of Hub Genes

**Figure 3:**
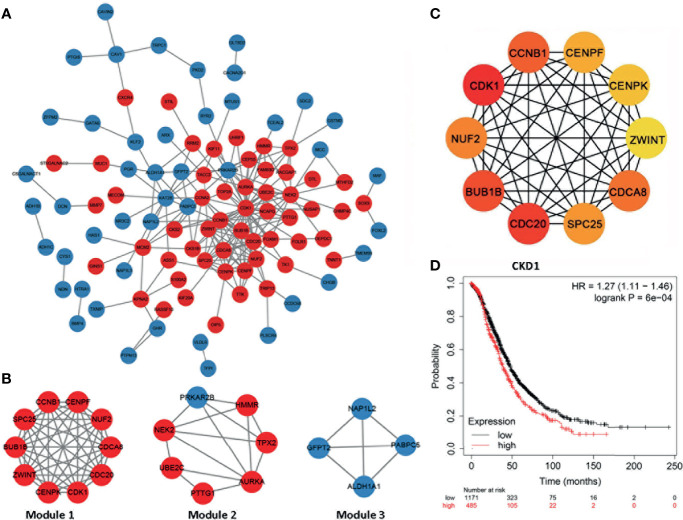
The protein–protein interaction network and hub genes. **(A, B)** EOC-related network; red indicates common upregulated genes and blue represents and common downregulated genes. The internal interactions between common DEGs were mined by the Metascape database, and the network was visualized using Cytoscape software. **(C)** Top 10 EOC-related hub genes. The network was analyzed by the cytoHubba plugin of Cytoscape software with the method of MCC. All the hub genes were upregulated in EOC tissues. **(D)** High CDK1 expression was correlated with poor prognosis of ovarian cancer patients (hazard ratio = 1.27, 95% CI: 1.11–1.46, p < 0.05).

Furthermore, we constructed the PPI network presented in **Figures 3A, B**, and the whole network was clustered in three modules by MCODE plugin of Cytoscape. Module 1 included all common upregulated genes and were enriched in cell division, chromosome segregation, and mitotic sister chromatid segregation, which were G2/M related. Module 2 consisted of common upregulated genes and one common downregulated gene, enriched in the regulation of mitotic cell cycle, cell cycle process, and mitotic cell cycle phase transition. Module 3 included all common downregulated genes but had no exact analysis results.

Next, hub genes were selected among the overlapping DEGs by the CytoHubba plugin of the Cytoscape. The top 10 genes were screened as hub genes, including CDK1, CDC20, BUB1B, CCNB1, CDCA8, NUF2, SPC25, CENPF, CENPK, and ZWINT in descending order (**Figure 3C**). CDK1 received the maximum score among them, and it was selected as the significant hub gene. CDK1 expressed a significantly higher level in ovarian cancer tissues, compared with normal tissues (**Supplementary Table 1**). Further, a higher expression level of CDK1 was correlated with poor prognosis of ovarian cancer patients (**Figure 3D**).

### EOC-Associated Drugs and GSEA

**Figure 4:**
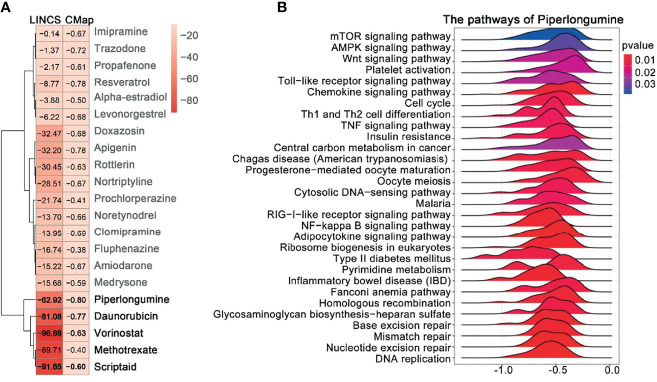
Prediction of drug candidates and drug GSEA analysis. **(A)** Similarity score table for the drugs having at least one significant association with connectivity map databases. Each row corresponds to a drug, and columns correspond to two-generation connectivity map databases. The score labels with numbers indicate the significance of the results. The row labels written in bold indicate the drugs we selected for further analysis. **(B)** GSEA analysis of the piperlongumine was assessed by the clusterProfiler package; p-value < 0.05 was considered statistically significant.

The overlapping DEGs generated for EOC were used to query CMAP and LINCS, respectively. By integrating the drugs from the two databases with score < 0, and p value < 0.05, we found that the 21 drugs were segregated into two clusters (**Figure 4A**); we selected the five drugs (piperlongumine, doxorubicin, vorinostat, methotrexate, and scriptaidin) in cluster 2, which had lower scores in both databases regarded as the potential drugs. Among them, four drugs (doxorubicin, vorinostat, methotrexate, and scriptaidin) have been used to treat EOC in clinical practice or clinical trials; PL received the lowest connectivity score, and there is litter evidence that it can treat EOC (23). Moreover, for piperlongumine, a total of 28 pathways were enriched (**Figure 4B**), including DNA replication, nucleotide excision repair, mismatch repair, and homologous recombination, which were closely related to the mechanism of EOC proliferation and drug resistance. Hence, we regarded PL as the candidate drug.

### Interactions Between Drug Candidate and Hub Gene

**Figure 5:**
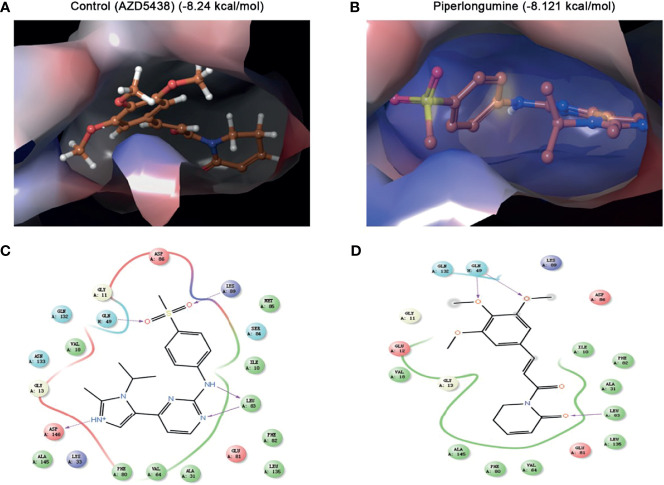
Molecular docking simulation for piperlongumine and CDK1. **(A, B)** Docked structure and interactions of drugs [**(A)** AZD5438 (control), **(B)** piperlongumine] binding to CDK1. **(C, D)** 2D interaction diagrams of the residues of CDK1 involved in the binding of drugs [**(C)** AZD5438 (control), **(D)** piperlongumine]. The Schrodinger Glide docking protocol was used for this analysis.

To further predict whether PL could be a direct CDK1 inhibitor, we performed molecular docking using the Schrodinger Glide docking protocol. Surprisingly, we found that PL showed a good binding affinity for CDK1 protein with the docking glide score of –8.121 kcal/mol, which is close to that of the known CDK1 inhibitor, AZD5438 (–8.24 kcal/mol). Most of the drugs appeared to have an equivalent glide score range from –8.121 through –2.662 kcal/mol. As displayed in **Figure 5**, the top scoring ligands, such as PL, were observed to interact with three residues Leu-83, GLN-132, and GLN-49 through hydrogen bonding with their side chains. Taken together, our data indicated that PL can bind to a similar pocket on CDK1.

### *In Vitro* Studies

**Figure 6:**
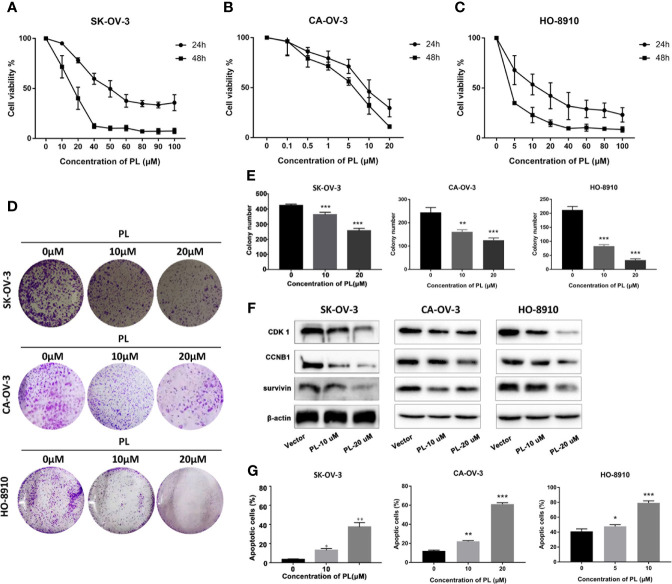
PL inhibited growth and induced apoptosis *via* targeting CDK1 in EOC cells. **(A–C)** Cell viability was assessed by MTT assay at 24 h and 48 h after treatment, and IC50 was computed accordingly. **(D, E)** Cell proliferation capacity was evaluated by colony formation assay, where cells after treatment were plated with a density of 200 cells/well and grown for 7 days followed by staining. **(F)** Expression levels of CDK1, CCNB1, and survivin proteins after treatment of PL at 10 or 20 µM for 24 h in EOC cells by Western blot analysis. β-Actin was used as an equal loading control for normalization. **(G)** Apoptotic cells were evaluated by Annexin-V and PI staining and analyzed by flow cytometry. All data are shown as mean ± standard deviation (n = 3). One-way ANOVA with the multiple-comparison test was used to calculate statistics: *p < 0.05; **p < 0.01; ***p < 0.001.

SKOV3, CA-OV-3, and HO-8910 cell cultures were exposed to different concentrations of PL for 24 h or 48 h, and cell viability was determined by MTT assay. As shown in **Figures 6A–C**, PL decreased cell viability in a concentration- and time-dependent manner. The IC50 value of SK-OV-3 was 49.32 and 16.28 µM in 24 and 48 h, respectively. For CA-OV-3, the IC50 in 24 and 48 h was 18.76 and 11.58 µM. For HO-8910, the IC50 in 24 and 48 h was 12.70 and 6.80 µM, respectively. Subsequently, a colony formation assay was also carried out; PL exposure caused a dose-dependent reduction in the number and size of colonies formed, compared with the control (**Figures 6D, E**). These data supported the inhibitory role of PL in ovarian cancer cell growth and colony formation. Additionally, PL induced lower levels of CDK1 and CCNB1 in a concentration-dependent manner, which is necessary for G2/M phase transitions of the cell cycle (**Figure 6F**). These results suggested that PL could inhibit EOC cell proliferation and affect the expression of CDK1.

Furthermore, to determine whether apoptosis was involved in PL-induced cytotoxicity, SKOV3 cells exposed to PL were stained with Annexin-V/FITC followed by flow cytometric analysis (**Figure 6G**). We observed an increase in apoptosis to 37.6% and 53.4% at 20 µM after 24 and 48 h, respectively.

## Discussion

In the current study, using gene expression data, a cluster of drugs that could potentially treat EOC was identified. Firstly, by merging TCGA mRNA-seq datasets and GEO mRNA microarray datasets, we generated overlapping DEGs as EOC signatures. Then, by integrating CMAP and LINCS databases, we identified potential drugs with lower negative connectivity scores that could evidently reverse EOC signatures. Based on the literature, four of these drugs were previously used clinically to treat EOC either as first-line treatment or as agents in clinical trials. This implies that we successfully predicted a group of known EOC drugs, without any hint of advanced drug information, suggesting that the remaining drug (piperlongumine) that we identified also has a high likelihood of treating EOC. Piperlongumine has been reported to inhibit several cancers, and only one study focused on ovarian cancer (24). In our study, the potential application of PL in EOC was further explored with a molecular docking test and *in vitro* experiments.

By merging EOC datasets from TCGA and GEO, 247 genes in total were considered as overlapping DEGs in EOC, including 103 common upregulated genes and 144 common downregulated genes. Then, common upregulated genes were mainly enriched in G2/M transition of the mitotic cell cycle, and the p53 signaling pathway, which are deemed to be a crucial pathway in the development and metastasis of EOC (25). Also, CDK1 was selected as the significant hub gene from the PPI network. It is well known that the abrogation of CDK1-CCNB1 activity blocks mitotic entry and arrests cells at the G2 phase (4).

We computed both CMAP and LINCS datasets to identify novel EOC drugs. Although CMAP has achieved remarkable success (26), some of its limitations cannot be ignored. For example, only five human cancer cell lines were used, and not all small molecules were tested on all these. The other drawbacks included limitations of dosages and time points and that several small molecules were tested using a 10-mM concentration with a 6-h perturbation time point. What is encouraging, LINCS covers 72 human cell lines and various cellular perturbations, including 15,000 small molecule compounds and 5,000 genes (gene silencing and overexpression). So we leveraged the LINCS dataset to increase the reliability of CMAP (27). In this study, the drugs with negative connectivity score were expected to be inversely correlated with EOC. Therefore, we focused on the five drugs with lower connectivity score from both CMAP and LINCS datasets. Among them, doxorubicin has been used clinically to treat ovarian cancer primarily by the mechanism of topoisomerase inhibition (28, 29). Vorinostat and scriptaid, two HDAC inhibitors, have been tested in ovarian cancer-associated clinical trials (30, 31). Methotrexate has been used as maintenance therapy in patients with advanced ovarian carcinoma (32). However, there is little evidence for PL treating ovarian cancer. Moreover, GSEA analysis showed that PL correlated with DNA replication, nucleotide excision repair, mismatch repair, and homologous recombination, which are important mechanisms for EOC drug resistance.

The molecular docking test, based on structure to design and understand the specific molecular mechanism, plays an important role in discovering drugs. There are a lot of successful examples in academia and industry. For instance, Mohamed et al. found that hesperidin formed a stable complex with a Polo-like kinase-1 active site by the approach of docking (33), Yu et al. used molecular docking to validate that Prestwick-685 and menadione may be the new esophageal carcinoma drugs (34). In our study, we selected the Schrodinger Glide docking protocol (35) to precisely simulate the interaction patterns and illustrate how PL acts on CDK1 proteins in the human body. Surprisingly, the results from docking tests demonstrated that PL could recognize and interact with CDK1 protein with a docking score of –8.121 kcal/mol, which was close to that of the known CDK1 inhibitor, AZD5438 (–8.24 kcal/mol). Therefore, it is suggested that PL has a considerable prospective role in the treatment of EOC by suppressing CDK1 proteins.

Piperlongumine, a biologically active alkaloid isolated from the roots of long pepper, is widely used as a traditional medicine in Ayurvedic medicine (36). It has been reported that PL selectively induces tumor cells death and delays tumor growth in hematologic tumors (37, 38) and diverse solid tumors (39–41). Furthermore, recent studies indicated that PL synergizes with cisplatin or paclitaxel to inhibit the growth of both chemoresistant and chemosensitive ovarian cancer cells (24). Currently, the cytotoxic effect of PL depends on the increase in reactive oxygen species (24, 42) and induction of apoptosis and autophagy, restoration of mutant p53, and cell cycle arrest (43–45). However, the arrest of G2/M triggered by PL was only detected as a phenotype. Hence, it needs a precise study to uncover how PL influences the cell cycle.

Due to the central role of CDK1 in the regulation of the G2/M phase, targeting CDK1 has emerged as a highly promising therapeutic strategy. Currently, several CDKIs have been investigated in clinical trials for treatment of various types of malignancies. For example, AZD5438, a CDK1/2 inhibitor, preferentially targets proliferating cells and typical chemosensitivity or radiosensitivity modulators (7, 46). However, the lack of inhibitor specificity currently limits clinical development. In our study, we predicted the possibility of PL being a novel selective CDK1 inhibitor. Mechanistically, in our study, PL inhibited cell viability in a dose- and time-dependent manner and induced apoptosis in ovarian cancer cells. In addition, PL led to decreased levels of the proteins CDK1 and cyclin B. Therefore, our study defines the details of PL which may target CDK1 to inhibit EOC.

In summary, we screened the DEGs from both two sources (TCGA and GEO), repurposed drugs by the two-generation CMAP database (CMAP and LINCS). Then, molecular docking and *in vitro* experiments were performed to explore and validate the drug–target interactions. However, there are also certain limitations; we only selected one ovarian cancer cell line and had no *in vivo* test to validate the exact function of PL to ovarian cancer. Therefore, to some extent, we utilized both *in silico* and *in vitro* experiments to predict that PL could be a novel drug to treat EOC. Furthermore, the results need more further research, as well as *in vivo* experiments.

## Data Availability Statement

The original contributions presented in the study are included in the article/**Supplementary Material**. Further inquiries can be directed to the corresponding authors.

## Author Contributions

YiZ and YeZ designed research; JB and EL performed bioinformatics research; DZ, CY, and ZJ performed cell research; JL, ZW, and MX sorted data; DZ, and LJ wrote the paper and revised the manuscript. All authors contributed to the article and approved the submitted version.

## Funding

This work was supported by the National Natural Science Foundation of China (Nos. 82073244, 81270036, 30901736), the Plan to Focus on Research and Development from the Science and Technology Project of Liaoning Province (No. 2017225029), and Shenyang Youth Science and Technology Innovation Talent Project (RC200267).

## Conflict of Interest

The authors declare that the research was conducted in the absence of any commercial or financial relationships that could be construed as a potential conflict of interest.

## Publisher’s Note

All claims expressed in this article are solely those of the authors and do not necessarily represent those of their affiliated organizations, or those of the publisher, the editors and the reviewers. Any product that may be evaluated in this article, or claim that may be made by its manufacturer, is not guaranteed or endorsed by the publisher.

## Abbreviations

CDK1, cyclin-dependent kinase 1; CMAP, Connectivity Map; DEGs, differentially expressed genes; EOC, epithelial ovarian cancer; GEO, Gene Expression Omnibus; GEPIA: the Gene Expression Profiling Interactive Analysis; GO, gene ontology; GTEx, the Genotype-Tissue Expression; KEGG, Kyoto Encyclopedia of Genes and Genomes; PPI, protein–protein interaction; PL, piperlongumine; TCGA, The Cancer Genome Atlas.
