# Supplementary material for: Identification of Novel Drug Candidate for Epithelial Ovarian Cancer via In Silico Investigation and In Vitro Validation
Source: Front Oncol. 2021 Oct 21;11:745590. doi: 10.3389/fonc.2021.745590 (PMC8568458; doi:10.3389/fonc.2021.745590)
Supplement: Supplementary file 1 [file Table_1.docx]

| **Downregulated Genes** | TXNIP RERG FRY ANKRD29 ITLN1 DPYD PDGFD RNASE4 AOX1 MTUS1 EFEMP1 ABCA8 MUM1L1 MAGI2-AS3 OGN CELF2 C21orf62 PDE8B SEMA3C PLCL2 WNT2B CLIP4 PMP22 CSGALNACT1 NBEA DFNA5 HLF ADH1C GPRASP1 ADH1B PPM1K GHR CAV2 PLEKHH2 TMOD2 PDE7B TMEM255A LHX9 CAV1 MAF HBB TCEAL7 PROS1 PKD2 HAND2-AS1 VIM CLEC4M GSTM3 NDNF ATP10D CHGB SFRP1 SNCAIP CNRIP1 FAM13C DMD SDC2 GPM6A DAPK1 MGARP SMTNL2 ZFPM2 ARMCX1 GIPC2 GFPT2 TSPAN5 GATA6 PGR PLCE1 MAOA TFPI CYBRD1 BAMBI ALDH1A1 PRKAR2B SMAD9 MYZAP NAP1L3 CXorf57 VGLL3 OMD RPRM CCDC80 KCNT2 PAPSS2 CLDN15 AMHR2 CPED1 CYP39A1 ARHGAP44 HTRA1 SDPR CMAHP TCEAL2 GNAI1 BCO2 MEOX2 PHLPP2 MEIS2 RARRES1 PSD3 PLSCR4 ARX ABI3BP TCF21 KLF4 NR3C2 HSD17B6 CLK1 SNAI2 KAT2B NDN VLDLR FABP4 SATB1 ECM2 CFH PRRX1 CHN2 B3GALT2 PRSS35 OLFML1 NAP1L2 NUDT11 TLE4 MRGPRF KLF2 EPS8 CCDC68 LAMA4 INTU FAT4 RP1-78O14.1 CALCRL KCNJ8 TRPC1 TMEM98 TSPAN8 CTNNAL1 RFPL1S CYS1 PTGIS DCN L3MBTL3 SESN1 INMT PDGFRA NR0B1 ZNF300P1 CACNA2D1 BEX1 AGTR1 LRRN4CL PEG3 HOXC6 KLHDC8A PCOLCE2 CDH11 TMEM176A DIRAS3 SLFN11 ZSWIM5 NEFH NT5E RYR2 MGP PON3 NLGN4X LINC00473 CLMP SIGLEC11 ID3 ADAMTS3 HAS1 STAR C7 GIMAP2 ASTN1 IGSF11 COL21A1 HHIP-AS1 |
| --- | --- |
| **Upregulated Genes** | WFDC2 FOLR1 CD24 EPCAM KLK7 ELF3 KLK8 KLK6 LYPD1 CP MMP7 UBE2C UCP2 SCGB2A1 ESRP1 MUC1 CDC20 DEFB1 TNNT1 TMPRSS4 KIAA0101 PTTG1 TPX2 EHF SOX17 C1orf106 CXXC5 TOP2A MAL RRM2 BIRC5 PSAT1 CKS2 FOXM1 BCL11A CDH6 KLHL14 SFN S100A2 KIF20A MECOM SLC2A1 CENPF NEK2 FAM83D CEP55 CDK1 PRAME ST6GALNAC2 CXCR4 SLC4A11 CDCA8 MKI67 MELK CHMP4C KIF2C CTHRC1 ZWINT TTK SYNE4 MPZL2 GLDC NCAPH NUF2 ASS1 SPON1 AIF1L TRIP13 SYTL1 HMMR RACGAP1 KIF18B RAD51AP1 SPC25 RASSF10 FAM64A EPHX4 NCAPG CDC45 ESPL1 BUB1B PPP1R14B ZBTB42 KIF4A AURKA PTH2R DTL CDCA3 ST6GALNAC1 DEPDC1 UHRF1 GRAMD2 GINS1 MTHFD2 STON2 NUSAP1 RMI2 NRTN RNASEH2A SLC52A2 ESM1 STIL CENPK ORC6 MCM10 HDGF PSRC1 FJX1 PRC1 NEIL3 EZH2 SAC3D1 PTX3 SCRIB RGS1 |
